# Early microstructural white matter changes in patients with HIV: A diffusion tensor imaging study

**DOI:** 10.1186/1471-2377-12-23

**Published:** 2012-05-01

**Authors:** Bianca Stubbe-Drger, Michael Deppe, Siawoosh Mohammadi, Simon S Keller, Harald Kugel, Nora Gregor, Stefan Evers, Peter Young, E-Bernd Ringelstein, Gabriele Arendt, Stefan Knecht, Ingo W Husstedt

**Affiliations:** 1 Department of Neurology, University of M uumlnster, Albert-Schweitzer-Campus 1, M uumlnster, D-48149, Germany; 2 Department of Radiology, University of M uumlnster, M uumlnster, Germany; 3 Department of Neurology, University of D uuml uuml sseldorf, D uumlsseldorf, Germany; 4 Department of Clinical Neuroscience, Institute of Psychiatry, King’s College London, London, UK; 5 Wellcome Trust Centre for Neuroimaging, UCL, Institute of Neurology, University College London, London, UK

**Keywords:** Depression, HIV-associated neurocognitive disorder, Fractional anisotropy, Neuropsychology

## Abstract

**Background:**

Previous studies have reported white matter (WM) brain alterations in asymptomatic patients with human immunodeficiency virus (HIV).

**Methods:**

We compared diffusion tensor imaging (DTI) derived WM fractional anisotropy (FA) between HIV-patients with and without mild macroscopic brain lesions determined using standard magnetic resonance imaging (MRI). We furthermore investigated whether WM alterations co-occurred with neurocognitive deficits and depression. We performed structural MRI and DTI for 19 patients and 19 age-matched healthy controls. Regionally-specific WM integrity was investigated using voxel-based statistics of whole-brain FA maps and region-of-interest analysis. Each patient underwent laboratory and neuropsychological tests.

**Results:**

Structural MRI revealed no lesions in twelve (HIV-MRN) and unspecific mild macrostructural lesions in seven patients (HIV-MRL). Both analyses revealed widespread FA-alterations in all patients. Patients with HIV-MRL had FA-alterations primarily adjacent to the observed lesions and, whilst reduced in extent, patients with HIV-MRN also exhibited FA-alterations in similar regions. Patients with evidence of depression showed FA-increase in the ventral tegmental area, pallidum and nucleus accumbens in both hemispheres, and patients with evidence of HIV-associated neurocognitive disorder showed widespread FA-reduction.

**Conclusion:**

These results show that patients with HIV-MRN have evidence of FA-alterations in similar regions that are lesioned in HIV-MRL patients, suggesting common neuropathological processes. Furthermore, they suggest a biological rather than a reactive origin of depression in HIV-patients.

## Background

Neuropathological examinations show white matter (WM) involvement in the brains of asymptomatic HIV-1 patients [1]. Diffuse WM pallor is the most frequent neuropathological feature of HIV-1 infection and has been found to be more prevalent and severe in the advanced stages of the disease [2]. HIV-1 infected magrophages and multinucleated giant cells preferentially invade the WM of the cerebral hemispheres, corpus callosum and internal capsule [3]. Several studies have reported widespread intra- and inter-individual regional HIV-1 RNA levels in the brain, and a predominance of productive HIV infection within the basal ganglia, brainstem and deep cerebral WM [4,5].

Standard structural magnetic resonance imaging (MRI) scans are unreliable for detecting HIV-induced WM alterations [6]. Diffusion tensor imaging (DTI) is more sensitive in detecting subtle WM changes relative to structural MRI scans [7-9]. Localized fractional anisotropy (FA) alterations are common findings in patients with HIV, which have been preferentially observed in frontal, callosal and deep WM regions [10-18]. DTI studies have also reported correlations between alterations in WM FA and cognitive impairment in patients with HIV [13,15,17-20]. In particular, Ragin et al. (2004b) reported that whole brain FA was reduced in patients with HIV and was significantly associated with severity of dementia [19]. The same authors later reported significant relationships between measures for putamen and visual memory, working memory, caudate (FA) and visual memory, and other regional and global correlates of FA [15]. Furthermore, Wu et al. (2006) reported associations between WM alterations of the splenium and dementia severity (decreased FA) and motor speed (decreased FA) [17].

The positive effect of HAART on HIV induced cerebral lesions has already been shown in conventional MRI [21]*.* If WM changes are of predictive value for the development of clinical symptoms such as HIV-associated neurocognitive disorder, motor impairment or depression, and as soon as these changes could be detected in patients prior to the onset of clinical symptoms, then this would have implications for the treatment of these patients. One way to assess the potential prognostic utility of DTI for the treatment of patients with HIV is to compare WM alterations between patients with lesions on MRI and those with no observable abnormality on MRI. In taking this approach, our study had two primary goals. Firstly, we determined white matter alterations in HIV patients with and without observable lesions on MRI relative to healthy controls in order to determine whether DTI is able to detect common WM microstructural damage in both patient groups. A common distribution of WM abnormalities in these patient groups would suggest that early identification of WM microstructural abnormalities in the absence of macroscopic lesions might have prognostic value and inform treatment strategies. Secondly, we sought to investigate FA differences between HIV patients with and without cognitive and affective impairment to clarify the significance of FA alterations of the brain for the development of HIV associated brain dysfunction and to get an insight in their multifactorial nature.

## Methods

### Participants

**Figure 1:**
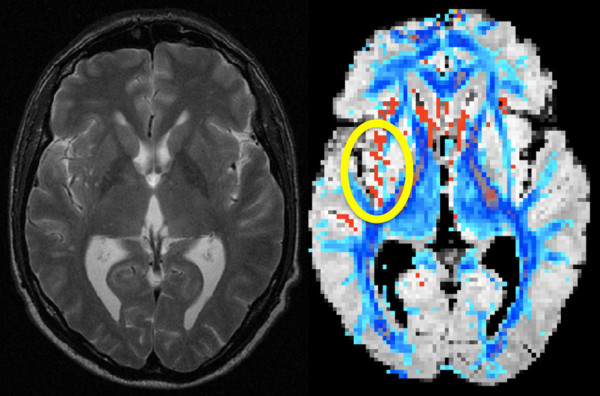
**Example of typical macrostructural WM alterations on conventional structural MRI (A) and the correspondent FA changes (B).** A: T2w image of one patient showing mainly unilateral hyperintense macrostructural lesions. B: Computer reconstructed hyperintensities of the same T2w dataset (red) overlaid with the patients FA (blue). Only FA values > 0.2 are shown. Close to the lesion (yellow circle) the FA is reduced (FA < 0.2) in comparison to the contralateral side. Similarly, uni- or bilaterally highly significant FA reductions were found in patients with macrostrucural lesions, as well as in patients without macrostructural WM hyperintensities.

The Ethics Committee of the Medical Faculty of the University of M uumlnster approved the study. All data was collected with the understanding and written consent of each patient. All patients approved the disclosure of their patient report to the examiner. Patients were recruited through the Department of Neurology at the University Hospital of M uumlnster between November 2004 and January 2006. Exclusion criteria included age < 18, history of non-HIV related neurological disease, current or past opportunistic infections of the central nervous system (CNS), current alcohol or substance abuse and specific structural brain pathology on conventional MRI, including FLAIR, T1-weighted, or T2-weighted imaging, other than slight cortical atrophy and few unspecific intracerebral lesions. Twenty-two patients (1 female, 21 males) originally participated in the study. However, two were excluded due to extensive intracerebral lesions on MRI scans (1 female, 1 male), and one patient withdrew consent. Full demographic, clinical and medical information for the 19 patients investigated using DTI in the present study is provided in Table 1. All patients underwent blood tests and lumbar puncture. CD4 positive cells and the T4/T8 ratio, as well as the viral load in plasma and cerebrospinal fluid were determined. Total white cell count, IgG ratio and oligoclonal bands, and levels of amyloid beta protein in cerebrospinal fluid were analyzed. Of the 19 patients, seven were found to have unspecific mild macrostructural lesions on MRI (HIV-MRL, see Figure 1). The remaining twelve patients had no observable abnormality on MRI (HIV-MRN). As control group we recruited 19 age-distribution-matched healthy control subjects (four female, 15 male). Age ranged between 26 and 56 years for patient and controls groups (patients: mean age 40.74 +/− 8.48 SD; healthy controls mean age 41.0 +/− 11. 86 SD; two-sample *t*-test: t = 0.079, df = 36, p = 0.9389).

**Table 1:** Clinical data of patients

**subject**	**age**	**Encephalo-pathy**	**Depression**	**years of HIV1 infection**	**medication**	**start of medication**	**VLcsf**	**VLblood**	**Cells of csf**	**CDC**	**CD4**	**abetaam**	**comment**
1	38	no	no	14,00	HAART	lw	50	50	1	B3	349	1.087	
2	41	no	yes	2,00	HAART	ew	unknown	unknown	unknown	C2	unknown	unknown	
3	29	?	yes	11,00	HAART	lw	50	50	2	B2	896	1.018	previous drug abuse
4	39	no	yes	0,50	no	en	46.220	9.296	19	B2	356	756	previous drug abuse, hepatitis C
5	40	yes	no	3,00	HAART	lw	50	50	0	C2	1.198	1.020	
6	35	no	yes	0,16	no	en	50.979	unknown	13	B1	1.251	unknown	
7	33	no	yes	4,00	HAART	ew	50	50	8	A1	595	1.018	
8	36	no	no	0,08	no	en	50	53	1	B1	1.331	857	hepatitis B
9	51	yes	?	15,00	HAART	en	50	50	0	B2	392	1.415	
10	50	no	no	2,00	no	en	unknown	28.000	9	B2	268	unknown	
11	44	no	no	2,00	HAART	ew	50	50	0	B2	574	1.228	
12	46	yes	no	9,00	HAART	lw	50	50	14	C3	500	0	
13	25	?	no	9,00	HAART	lw	50	50	11	B3	452	1.220	previous drug abuse, hepatitis C
14	29	no	no	2,00	HAART	ew	50	50	3	A2	186	359	
15	55	yes	yes	9,00	HAART	lw	50	50	0	B2	1.031	unknown	
16	40	no	yes	10,00	no	en	9.148	3.728	20	B2	535	916	hepatitis C
17	38	yes	yes	3,00	no	en	114	5.162	8	A2	474	730	
18	40	yes	no	17,00	HAART	lw	245	5.808	3	C3	120	700	
19	56	yes	yes	16,00	HAART	ew	50	110	4	B2	721	unknown	

? = testing not clear; lw = late with medication, ew = early with medication, en = early without medication; VL = virus load; csf = cerebrospinalfluid; HAART = Highly Active Anti-Retroviral Therapy Highly Active Anti-Retroviral Therapy.

For classification see for example http://www.aids-ed.org/aidsetc?page=cg-205_hiv_classification.

Prior to the study, each patient underwent neurological and detailed neuropsychological examinations including the Memorial Sloan Kettering Scale for AIDS Dementia Complex, the Unified Parkinson Disease Rating Scale, neuropsychological tests of verbal, visual and working memory, tests for constructions and psychomotor skills, the frontal system and literacy. A neuropsychologist, who was not informed of the MRI results, assessed depression status as well as evidence for HIV associated cognitive disorder [22-29]. Furthermore, indicators of motor functions were also recorded, which included simple reaction times and contraction time of most rapid single index finger contractions, postural tremor of outstretched hands and frequency of most rapid alternating finger movements, each for the right and left hand [30]. Information regarding duration of the disease, co-infection with hepatitis, previous drug abuse and medication were collected (see Table 1).

### Magnetic resonance imaging

All MRIs have been acquired on a Philips Intera 3 T scanner (Best, The Netherlands). Structural images were acquired by 3D T1w (1.0 x 1.0 x 1.0 mm^*3*^*), T2w, and FLAIR sequences*. For DTI we used echo planar imaging (EPI) with 20 diffusion directions (two b-factors, 0 s/mm^2^ and 1000 s/mm^2^, TR = 9.8 s/TE = 95 ms, acquisition matrix: 128 x 128, 36 axial slices, voxel size: 1.8 x 1.8 x 3.6 mm^3^ (reconstructed to 0.89 x 0.89 x 3.6 mm^3^), 2 averages, scanning time 7:46 min). All DTI image processing was performed with the “M uumlnster Neuroimaging Evaluation System (EVAL)” employing the recently created sophisticated registration toolbox that was developed for optimal image processing for voxel based statistics (VBS) [31,32] and a new, 3D eddy current correction approach [33]. All time consuming calculations were carried out on a 64-bit 64-processor parallel computer (Sun Microsystems, Inc., Palo Alto). Briefly, the registration toolbox provides multi-contrast registrations steps (contrasts: FA and b0), which are automatically calculated iteratively. Prior to the iterative registration the EPI images measured without diffusion gradient (b0 images) are registered to the to the statistical parametric mapping (SPM, http://www.fil.ion.ucl.ac.uk/spm/) EPI template using affine transformations to create a customized common registration template. The multi-contrast registration is then iteratively applied to obtain normalized FA images. The optimized registration technique includes also an individual hemisphere registration. The latter is achieved by setting the pixel values in the unconsidered hemisphere to zero, and in a separate step, registering the previously unconsidered hemisphere by flipping the original image and repeating the registration process. The output of all calculated FA images correspond to the MNI coordinate space. The combination of improved eddy current correction and iterative multi-contrast registration enables the detection of very focal alterations in water diffusion parameters [9].

**Figure 2:**
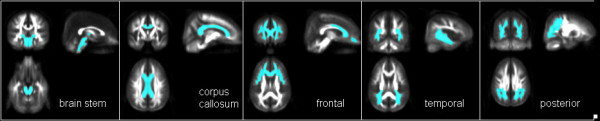
**Regions of interest as a priori defined.** 1) the frontal subcortical WM bilaterally 2) the temporal subcortical WM bilaterally 3) the posterior subcortical WM bilaterally 4) the corpus callosum bilaterally and 5) the WM of the brainstem *(from left to right)*.

We defined five regions of interest (ROIs) a priori to assess the specificity of regional FA alterations: 1) frontal lobe WM bilaterally 2) temporal lobe WM bilaterally 3) posterior WM bilaterally, including the parietal and occipital lobes 4) the corpus callosum and 5) the WM of the brainstem. The anatomical locations of these regions are indicated in Figure 2. To define the position of the ROIs as objectively and reproducibly as possible within each of the individual brains, each ROI was defined by a mask that was also registered into the MNI space. Mean FA values of all five ROIs were calculated for all patients and controls. As established in a previous study, this method provides robust, sensitive and observer-independent results [34]. In a previous study we could detect age dependent differences on FA but no differences between gender [35]*.*

Group differences in ROI-specific mean FA values were evaluated using a multiple measurement ANOVA with the repeated factor “Region” followed by appropriate post-hoc tests. Analysis of subgroups (Hep C-infected, previous drug abuse) were performed with t-tests for independent samples.

Voxel-by-voxel FA differences between patients and controls were evaluated using SPM2 on images that were smoothed by an 8 mm isotropic Gaussian kernel. Differences of FA values among groups were statistically evaluated by analysis of variance, (*p* < 0.05, corrected; minimum cluster size 100 voxels). For figures we used more liberal (uncorrected) thresholds to show the extent of WM alterations, but all illustrated clusters achieved significance at corrected statistical thresholds. Relationships between WM changes and motor and laboratory test parameters were analyzed using Persons product moment correlations.

## Results

### ROI analysis

**Figure 3:**
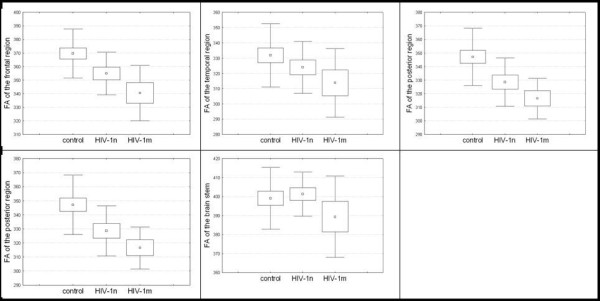
**FA values per group.** FA x 1000 of all priori defined ROIs for the healthy controls, the patients with normal conventional MRI and the patients with mild alterations on conventional MRI with means, standard errors and standard deviations.

The means and standard deviations of the ROI based FA values are presented in Table 2 and illustrated in Figure 3. Analysis of variance with Greenhouse Geisser correction, the repeated factor “Region” (corpus callosum, brainstem, frontal lobe, temporal lobe, and posterior lobe), and the group factor “Group” (all patients versus control subjects) revealed that patients had significantly decreased FA compared to healthy controls in the corpus callosum (t = −2.301, df = 36, p = 0.027), the temporal (t = −3.391, df = 36, p = 0.002) and posterior region (t = −3.647, df = 36, p = 0.001). A similar trend was seen for the frontal lobe (t = −1.786, df = 36, p = 0.083). Further, the analysis of variance showed a significant Group x Region interaction (F = 3.72, d.f. = 4, p = 0.034). An ANOVA considering the effect of lesion status (HIV-MRN, HIV-MRL and controls) revealed a significant Group x Region interaction (with Greenhouse Geisser correction F = 6.97, df = 8, p < 0.001). Post-hoc testing revealed that FA of the corpus callosum was significantly decreased in patients with HIV-MRL compared to the HIV-MRN group (t = 3.94, df = 17, p = 0.01). For the HIV-MRL group, the FA of the corpus callosum, temporal lobe and posterior lobe was significantly decreased compared to healthy controls (all t’s ≤ 3.49, df = 24, all p’s ≤ 0.01). Again, a similar trend was seen for the frontal lobe (t = 1.92; df = 24, p = 0.067).

**Table 2:** Fractional anisotropy means plusmn SD by group

**Brain region**	**HIV-1 n (n = 12)**	**HIV-1 m (n = 7)**	**Control (n = 19)**
Frontal lobes	0.324 plusmn 0.0017	0.314 plusmn 0.0022	0.332 plusmn 0.0021
Temporal lobes	0.355 plusmn 0.0016	0.341 plusmn 0.0021	0.370 plusmn 0.0018
Posterior lobes	0.329 plusmn 0.0018	0.317 plusmn 0.0015	0.347 plusmn 0.0021
Corpus callosum	0.415 plusmn 0.0028	0.350 plusmn 0.0044	0.422 plusmn 0.0037
Brainstem	0.401 plusmn 0.0012	0.389 plusmn 0.0021	0.399 plusmn 0.0016

There was no difference in FA between patients with and without HIV-associated neurocognitive disorder or patients with and without neuropsychological evidence for depression as revealed by multiple measurement ANOVA. There was no correlation between the duration of HIV infection and the FA, and there were no FA differences between patients taking HAART or those who not. Furthermore, the onset of medication in the time-course of HIV-1 infection (early with medication, late with medication, early without medication) had no influence on the FA. Unpaired t-tests revealed no differences in FA between HIV-1 positive patients with and without previous drug abuse or, with or without hepatitis C infection, respectively. For laboratory parameters, there was a correlation between the FA of the brainstem and the cell count of the central spinal fluid (CSF) (r = 5.07, p = 0.032). FA of the brainstem was correlated with CSF levels of amyloid beta protein (r = 0.534, p = 0.049). For the motor functions, the contraction time of most rapid single index contraction and the FA of the frontal lobe were correlated (r_ctri_ = −.485, p = 0.049/r_ctl_ = −.530, p = 0.029).

### Voxel-based statistics

**Figure 4:**
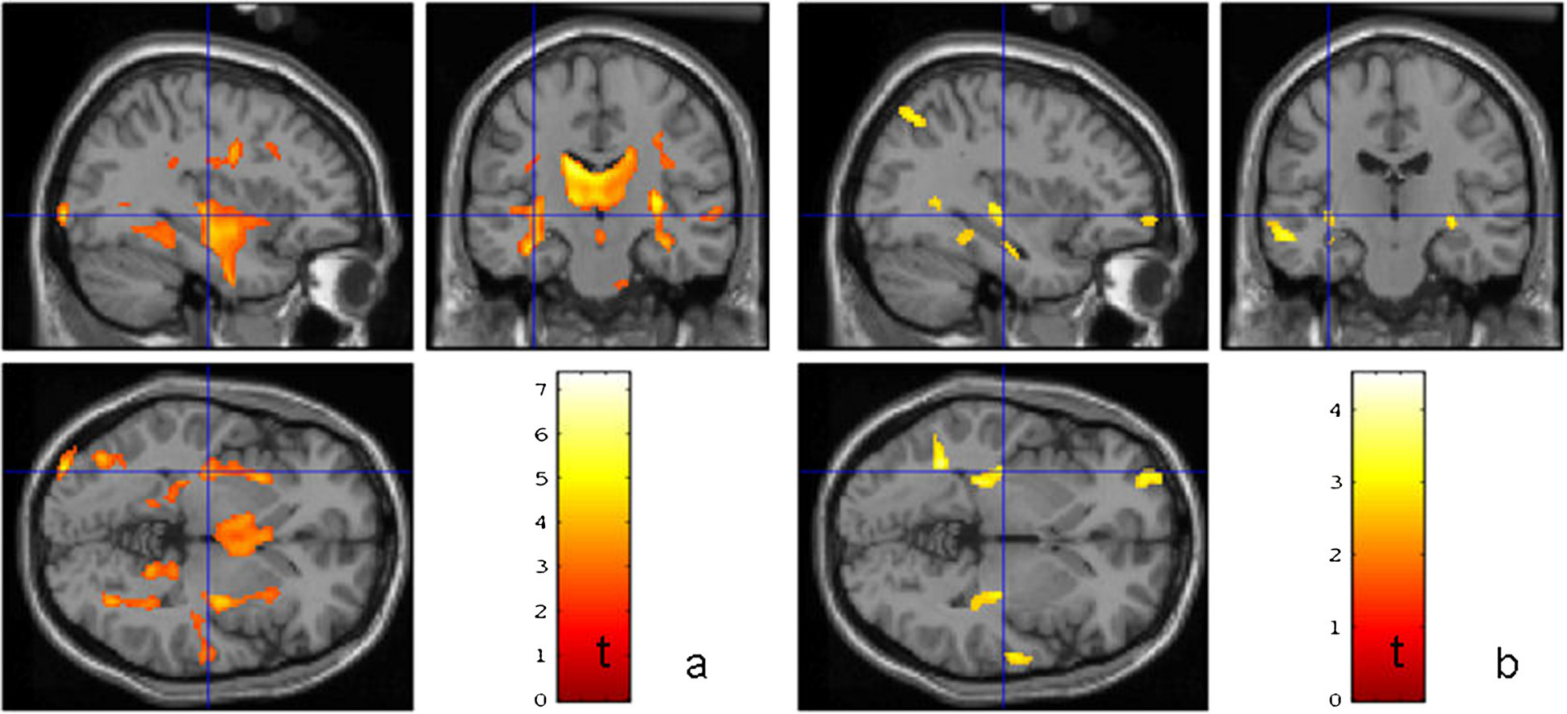
**Results of the SPM voxel-by-voxel group statistic for the MRI-groups Comparison between a) the seven patients with unspecific lesions in conventional MRI versus 19 healthy controls b) the 12 patients with no lesions in conventional MRI versus the same 19 healthy controls.** The lines represent corresponding cut planes of the sections (−34 mm,-18 mm,-2 mm; Montreal Neurological Institute space). The color coded *t-*values represent significant (*p* < 0.01) FA reductions in the patients compared to the controls (minimum cluster size: 100 voxel).

Significant and widespread FA reduction in the HIV-MRL group relative to the healthy controls was observed encompassing the inferior fronto-occipital and inferior longitudinal and uncinate fasciculi, the posterior limb of the internal capsula, cortico-spinal, cortico-pontine and cortico-bulbar tracts (p < 0.05, corrected, minimum cluster size 100 voxels). Relative to controls, patients with HIV-MRL had reduced FA in similar WM regions, but these effects were not as pronounced (Figure 4).

**Figure 5:**
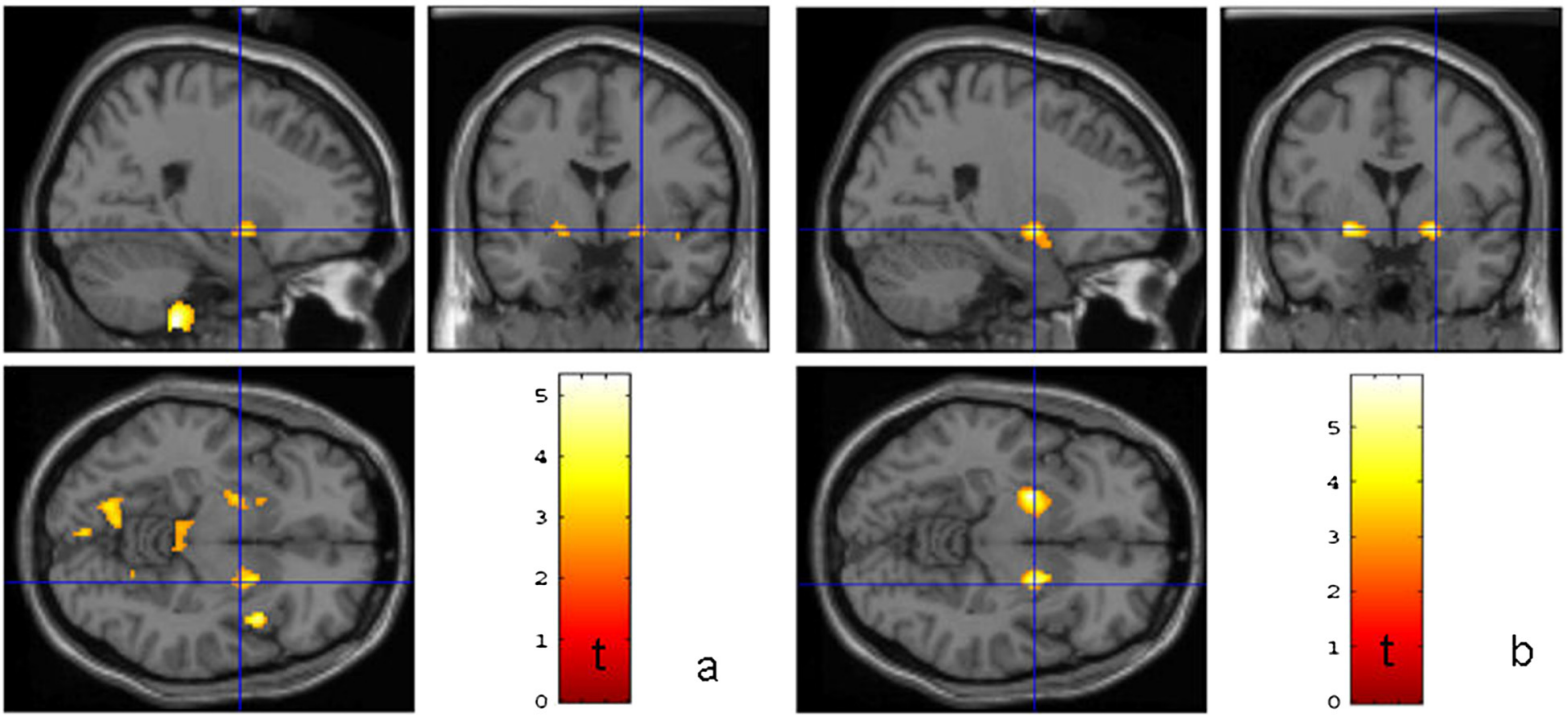
**Results of the SPM voxel-by-voxel group statistic for patients with and without depression.** Comparison between a) the nine patients with neuropsychological evidence for depression versus 19 healthy controls b) the *nine* patients with neuropsychological evidence for depression versus nine patients without. The lines represent corresponding cut planes of the sections (23 mm, -1 mm, -8 mm; Montreal Neurological Institute space). The color coded *t-*values represent significant (*p* < 0.01) FA reductions in the patients with evidence for depression compared to the controls (minimum cluster size: 100 voxel).

Patients with neuropsychological evidence *of* HIV associated neurocognitive disorder had significant widespread FA reduction without preference of a specific region (p ≤ 0.01) compared to HIV patients without *such* evidence. For HIV patients with neuropsychological evidence of depression, compared to HIV patients without, and healthy controls, significantly increased FA (p < 0.01, small volume corrected) was observed in the ventral tegmental area, nucleus accumbens and ventral medial prefrontal cortex in both hemispheres (see Figure 5).

## Discussion

Results presented here indicate that HIV-patients have DTI-derived microstructural alterations relative to healthy controls encompassing multi-lobar brain regions. Patients with HIV-MRL had substantial FA reduction, particularly adjacent to the observable lesions, and patients with HIV-MRN had less substantial FA reduction, but interestingly, in similar WM regions as HIV-MRL patients. Patients with neuropsychological evidence of depression showed bilaterally increased FA in the globus pallidus, ventral tegmental area and nucleus accumbens relative to patients without evidence for depression. Patients with evidence of HIV associated neurocognitive disorder showed an overall FA reduction relative to patients without evidence for HIV associated neurocognitive disorder.

### WM abnormalities in patients with HIV

There have been several studies published that have used DTI techniques to investigate WM abnormalities in patients with HIV [10-18,36]. Most of these studies have identified WM alterations in multiple brain regions, most notably the corpus callosum, deep WM regions and frontal lobe WM, which is consistent with the data presented here. However, replication of the precise regional WM alterations has been difficult between studies [36]. There are additionally differences in the nature of WM abnormalities, given that some studies have preferentially observed mean diffusivity alterations [13,36] whilst other studies report multiple FA changes. Contrary to other investigations, our study employ*s* a comparison among patients with HIV-MRN and HIV-MRL. We found comparable alterations in both groups, but more prominent alterations in HIV-MRL.

### WM alterations, cognitive impairment and laboratory parameters

Some DTI studies have reported correlations between alterations in WM FA and cognitive impairment in patients with HIV [13,15,17,19,20], whereas others reported no association [36,37]. Ragin et al. (2004b) reported that whole brain FA was reduced in patients with HIV and was significantly associated with severity of dementia [20]. The same authors later reported significant relationships between diffusivity and anisotropy of the putamen and verbal memory (mean diffusivity (MD)), visual memory (FA), working memory (FA), and overall cognitive impairment (MD), caudate FA and visual memory, and other regional and global correlates of FA and MD [15]. Wu et al. (2006) reported associations between WM alterations of the splenium and dementia severity (decreased FA) and motor speed (decreased FA and increased MD) [17]. In our sample, FA differences of the ROIs did not reached significance between HIV-1 positive patients with and without neuropsychological evidence of HIV-associated neurocognitive disorder. However, the FA of the frontal region was significantly associated with the time of index finger contractions, indicating motor slowing. Similarly, Cloak and collegues (2004) found increased frontal WM diffusion associated with motor slowing [38]. Minor motor deficits related to HIV-1 are a clearly defined preclinical manifestation of HIV-1. Motor slowing predicts progression in HIV associated brain disease [30]. Additionally, WM alterations detected on a voxel basis showed significant global and diffuse FA reduction in the HIV patient group with neuropsychological evidence for HIV-associated neurocognitive disorder compared to the HIV patient group without. This indicates that WM alterations in patients with HIV associated neurocognitive disorder do not have uniform structural patterns between patients, and can show very different individual pictures.

In our sample there was no association between CSF viral load and WM alterations. Furthermore, we could not detect an influence of the duration of HIV-1 infection as well as the onset of medication on WM alterations. This is in concordance with another study [36]. However, in the present study the viral loads were not extensively high (only two patients with about 50.000 HIV messenger RNA copies/mm³). Filippi and colleagues studied patients with viral loads greater than 400.000, and found WM alterations associated with viral load levels [39]. It is possible that higher viral load facilitates the detection of an association between CSF viral load and WM alterations.

Notably, FA of the brainstem showed a positive correlation with the cell count of CSF, and a negative correlation with the CSF levels of amyloid beta protein in HIV-1 positive patients. Different studies found a dominance of productive HIV infection within the brainstem [40,41]. Furthermore, HIV-1 positive patients with dementia have significantly decreased CSF levels of amyloid beta protein [42]. Further studies are needed to clarify the significance of FA alterations of the brainstem for the development of HIV associated cognitive disorder.

### WM alteration and depression in HIV patients

Patients with neuropsychological evidence of depression showed bilaterally increased FA of grey matter (GM) structures, including the ventral tegmental area, the nucleus accumbens and the globus pallidus. These alterations were observed in comparison with healthy controls, as well as in comparison with HIV patients without neuropsychological evidence of depression. Increased subcortical GM FA has been previously shown to correlate with normal aging [43,44] and is also found in Huntington disease [45,46].

Recent studies have shown that the nucleus accumbens and the ventral tegmental area contribute to the pathophysiology and symptomatology of depression and may even be involved in its aetiology [47,48]. Paul and colleagues found apathy, a discrete component of depression, to be associated with decreased volume of the nucleus accumbens in HIV infected men [49]. In simian immunodeficiency virus infected rhesus monkeys dopamine content was reduced in the nucleus accumbens compared to uninfected animals [50]. In this context, our results implicate a biological rather than a reactive origin of depression in HIV infected patients.

### WM alterations in HIV-MRL and HIV-MRN patients

Patients with HIV-MRL and HIV-MRN had multi-lobar alterations of mean FA. Patients with HIV-MRL had substantial FA reduction, particularly adjacent to the observable lesions, encompassing the inferior fronto-occipital and inferior longitudinal and uncinate fasciculi, the posterior limb of the internal capsule, cortico-spinal, cortico-pontine and cortico-bulbar tracts. Patients with HIV-MRN had less substantial FA reduction, but interestingly, in similar WM regions as HIV-MRL patients. To our knowledge this is a new finding. Autopsy studies reported WM lesions in asymptomatic HIV patients who died of unnatural causes [1,51]. Using DTI, Stebbins and collegues (2007) reported decreased WM alterations present even in radiologically defined normal appearing WM in non-demented, community-dwelling patients. Chang et al. found greater than age-related changes in brain diffusion of HIV patients after 1 year although no comparison was made with patients with visible lesions, [10,36]*.* Chen et al. (2009) identified widespread abnormal regions in HIV patients with and without dementia, although a greater distribution was observed in patients with dementia [11]. Gongvatana identifying changes in white matter tracts associated with more advanced HIV infection [52]). Our results indicate that HIV infection causes widespread WM alteration in both HIV-MRN patients and HIV-MRL patients. The presence of common WM alterations in HIV-MRN and HIV-MRL patients suggests that patients with HIV undergo a common neuropathological process. HIV-patients are at great risk for future cognitive impairments, dementia and cognitive decline. The possibility of detecting FA associations between clinical, laboratory and neuropsychological parameters will provide an insight into pathological interactions and underlying causes. Use of DTI in HIV patients may allow early prognostic monitoring of the disease and give implications for the treatment of these patients. The present results motivate further DTI-studies to investigate the potential of DTI as an indicator for early or enhanced antiretroviral medication.

## Conclusions

DTI is more sensitive to detect early microstructural WM alterations in HIV patients than structural imaging techniques. Patients with HIV-MRN show evidence of FA-alterations in similar regions that are lesioned in HIV-MRL patients, suggesting a common neuropathological process. These findings also indicate the potential prognostic power of DTI, which can detect WM alterations early in the course of the disease.

Results presented here also suggest a biological rather than a reactive origin of depression in HIV patients. Patients with HIV associated neurocognitive disorder show an overall FA reduction suggesting no uniform structural patterns between patients. The possibility of detecting FA associations between clinical, laboratory and neuropsychological parameters will provide an insight into the pathological interaction and underlying causes. The present results motivate further DTI-studies to investigate the potential of DTI as an indicator for early or enhanced antiretroviral medication.

## Competing interests

B.S., S.S.K., H.K., N.G., S.E., P.Y., G.A. I.H. reported no disclosure. M.D. and S.M. received support by the transregional Collaborative Research Centre SFB/TR 3 (Project A8) of the Deutsche Forschungsgemeinschaft (DFG). E.R. has received travel expenses and honorariums from Boehringer Ingelheim, Sygnis, Neurobiological Technologies, Novartis, Novo-Nordisc, Sanofi-Aventis, Solvay, Bayer Vital, M’s Science, Servier, UCB, Trommsdorff for serving as a member of Steering Committees, Safety Committees in clinical trials, and as a speaker and consultant. S.K. received travel expenses from Sanofi-Avensis, Boeringer-Ingelheim und Jansen-Cilag and research support from Interdisziplinery center of clinical research M uumlnster, Innovative medizinische Forschung M uumlnster, Deutsche Forschungsgemeinschaft and Marie Curie Research and Training network and from Volkswagen Stiftung.

This work was supported by the Neuromedical Foundation (Stiftung Neuromedizin), M uumlnster and the German Competence Network HIV/AIDS.

## Authors’ contributions

BS and MD contributed equally to this article. BS; MD; SM; SK and IH participated in study design. BS; HK; NG, SE;ER; PY; GA; IH contributed to data collection. BS; MD; SM; SSK; SK participated in data analysis, all in data interpretation, and BS; MD and SSK in writing of the report. All authors read and approved the final manuscript.

## Pre-publication history

The pre-publication history for this paper can be accessed here:

http://www.biomedcentral.com/1471-2377/12/23/prepub
